# Addressing Language Barriers in the Intensive Care Unit: A Case-Based Reflection and Brief Appraisal of the Literature

**DOI:** 10.7759/cureus.55646

**Published:** 2024-03-06

**Authors:** Vijayvardhan Kamalumpundi, Carolina Gonzalez Bravo, Daniel J Kohn, Patrick McGonagill, Kristina Guyton

**Affiliations:** 1 Internal Medicine, Roy J. and Lucille A. Carver College of Medicine, University of Iowa, Iowa City, USA; 2 Surgery, University of Iowa Hospitals and Clinics, Iowa City, USA

**Keywords:** language interpretation services, intensive care unit, communication barriers, cultural competency, patient safety

## Abstract

At present, a substantial number of individuals in the US face limited English proficiency (LEP), posing difficulties for healthcare providers. Language barriers between healthcare providers and patients can lead to poor quality of care, especially in patients with hyperacute conditions such as stroke, myocardial infarction, acute trauma, and more. In the intensive care unit (ICU), diagnosis and rapid treatment decision-making rely on taking an accurate patient history and physical exam. While in-person interpreters are the gold standard for patients with LEP, the fast-paced nature of the ICU may require alternate modes of using interpreting services to fit ICU workflows. We present a case-based reflection of a patient with LEP who presented to our ICU after a motor vehicle accident. We present this case from the perspective of a third-year medical student caring for a patient while rotating in an ICU service. We illustrate how language interpretation impacted the patient’s care. We conclude by appraising the ICU literature and providing solutions to addressing language barriers for ICU patients with LEP to deliver patient-centered, high-quality care.

## Editorial

Currently, 25 million people in the US have limited English proficiency (LEP) [1]. LEP is defined as a limited ability to read, speak, write, or understand English [2]. With rapidly changing language demographics in the US, patients are experiencing language barriers in many areas of society, including the healthcare system [1]. Language barriers can lead to errors in patient assessment and exacerbate disparities such as misdiagnosis, delayed treatment, poor patient understanding, and decreased patient and family satisfaction. Furthermore, limited patient understanding may lead to inadequate adherence to medical treatment, a key factor associated with increased hospital readmissions [1].

Minimal research has been conducted regarding language barriers in hyperacute settings such as the intensive care unit (ICU). Clear communication is critical in the ICU, especially in a setting where diagnostic decision-making relies heavily on a patient’s history and physical exam. Poor communication with the patient in the ICU setting hinders proper interpretation of a neurologic exam and prevents prompt treatment of emergent patient conditions (e.g., toxic ingestions and stroke workup), among others [1]. Furthermore, communication between healthcare providers and a patient’s family is critical to providing patient-centered care. In-person interpreters are ideal for establishing rapport and facilitating language interpretation and communication between the care team, the patient, and their family. However, the fast-paced nature of the ICU may require alternate modes of utilizing interpreting services to fit ICU workflows.

We present a short case-based reflection on a patient with LEP who was transferred to the ICU following a motor vehicle accident. This perspective is provided by a third-year medical student who rotated on our ICU service. We illustrate how language interpretation impacted the patient’s care. Finally, we conclude by appraising the ICU literature and suggesting alternative ICU workflows to help patients with LEP navigate language barriers, ensuring they receive equitable, patient-centered care.

Case-based reflection

“It was the third day of my ICU rotation. I remember six or seven infusion pumps dripping antibiotics, fluids, and vasoactive medications into a patient we were desperately trying to sustain. I was a part of the ICU care team caring for a middle-aged man who had been involved in a multiple-fatality motor vehicle accident.The patient had a prolonged extrication, and upon arrival at the emergency department, he was immediately intubated and sedated. An interpreter was not used to facilitate the intubation, as little was known about the patient’s identity at the time. He had suffered blunt polytrauma to the head, neck, chest, and lower extremities that required emergent procedures for stabilization and prompt transfer to the ICU.

The patient was from the Democratic Republic of Congo and spoke very limited English. The staff initially communicated with the patient’s friend, who arrived at the ICU, and disclosed that the patient spoke French. Every morning, the ICU attending and the trauma surgery team would examine the patient with minimal communication. The patient’s primary support was his wife, who also spoke limited English and was not usually present during morning rounds. The ICU and trauma surgery teams spent time on rounds thoroughly listening to and updating patient families, but calling an interpreter for the patient seemed like one extra step in their incredibly busy day. Multiple members from consulting teams would enter the patient’s room to round throughout the day, although an interpreter was seldom utilized. As the patient was weaned off sedation and extubated, he became more aware of his surroundings and would become intermittently combative. The healthcare team would ask, “Douleur, douleur, douleur?” in French (translation: “pain?”) as we pressed on different surgical incisions. After spending a few minutes in the patient’s room, the resident would inform the patient of the plan for the day in rushed English before they hurried off. It was unclear if the patient was able to comprehend the information.

At our hospital, psychiatric nursing is a service offered to help patients address their emotional response to their injuries or illnesses. Psychiatric nursing was consulted early on in the patient’s hospitalization and helped deliver difficult news about the fatalities of the accident. Notably, an in-person French interpreter was present with the psychiatric nursing staff when conversing with the patient and his family. After a month, the patient was discharged to an acute rehabilitation facility. He was appreciative of the care he received, especially from psychiatric nursing.”

Discussion

This experience is common in ICUs across the globe and in many other areas of medicine. Roughly 10-30% of patients with LEP receive professional interpretation services [2]. Although it is unclear whether the underutilization of interpreters affected our patient’s treatment decisions or clinical outcomes, it is certain that he had suboptimal communication with the ICU and surgical teams. Although the psychiatric nursing teams only visited the patient four times throughout his hospitalization, they built rapport with him that was evident on discharge. They were able to communicate with him and his family in a culturally responsive manner, which undoubtedly improved the patient’s experience with the care teams.

At the beginning of medical school training, we take an oath acknowledging our responsibility to our patients. Paradoxically, this sense of responsibility is often compromised by the stressors that are placed on providers by the healthcare system. The volume of patients per provider in combination with the lack of interpreting services are just a few barriers that directly conflict with providing optimal care. Ideally, a healthcare system should be designed to cater to the diverse needs of the patient population. When those systems fail in design, the care for the patient becomes compromised. In the case of our patient, it was the design of the system that failed the patient rather than the individuals who provided the care. Despite our intentional best efforts, the fast-paced nature of the ICU made it nearly impossible for the provider to request interpreting services in a timely manner. This is a shared experience that remains frequently unaddressed in the literature. We propose circumventing these issues using evidence-based strategies (Table 1) to facilitate communication between patients with LEP and their healthcare teams.

**Table 1:** Evidence-based strategies to better serve patients with LEP in the ICU EMR, electronic medical record; ICU, intensive care unit; LEP, limited English proficiency

Strategy	Potential benefits to patient care	Feasibility (financial, time, and personnel)
Increasing access to professional language services (in person or by telephone)	Using interpreters provides optimal care for patients with LEP [1]. Interpreters are trained to engage in conversation with medical vocabulary that allows accurate translation of medical jargon [1]. Professional interpreters provide an objective voice and ensure thorough interpretation of the conversation in a culturally responsive manner.	Both in-person and phone interpreters are made available by contract with external language interpreting agencies that are compliant with the Health Insurance Portability and Accountability Act. If a healthcare system receives federal funding, interpretation services are protected under Title VI of the Civil Rights Act and the Affordable Care Act Section 1557. The quality of interpretation by contracted interpretation services is evaluated using rigorous methodology. Rare languages are likely to be covered by interpreters using technology-based, professional interpreting services. Phone interpreters are usually available 24/7, making them an ideal tool for the ICU.
Integrating interpreter use into ICU rounds	Most of the engagement between care teams and families in the ICU occurs during rounds, lending a place where interpreters can be leveraged [3]. Clinicians can improve daily family engagement and communication by inviting family members to attend ICU rounds [2,3].	Scheduling interpreting services ahead of time (i.e., before rounds) can maximize the time that providers spend addressing patient or family questions. Scheduling an interpreter could be facilitated by the patient’s nurse or a designated team member (e.g., a medical student). For routine conversations or updates that take place during the day, a phone interpreter could be used. A badge card with instructions on how to page or phone an interpreter could be given to each healthcare provider.
Disseminating translated written information about various diagnoses and treatments into multiple languages	Written communication in conjunction with verbal communication between healthcare teams and patients’ families can be important to facilitate understanding of a patient’s condition [4].	Language-translated pamphlets detailing common diagnoses (e.g., stroke and myocardial infarction) and treatments may be disseminated to patients and their families. Many EMR systems have integrated translated information about common, disease-specific information that can be printed and given during rounds.
Highlighting patients’ needs for an interpreter in the EMR and during handoff	Visual aids serve to reduce the provider's cognitive overload by transmitting messages in a straightforward manner [5]. Having a dialogue box integrated into the EMR for identifying patients with LEP could greatly increase the use of interpreters by consulting services and other healthcare providers taking care of the patient.	A dialogue box in the EMR that identifies a patient’s LEP needs creates a user-centered approach that could be easily implemented in the ICU. Integrating interpreter needs during handoffs between providers could increase the uptake of interpreter use in patient care.
Training all healthcare staff on how to use interpreter services	Formal training regarding how to use interpreting services has been associated with higher self-efficacy in knowing when an interpreter is needed [2].	An e-learning module detailing how to access and use interpreting services could be implemented for all new hires in the ICU or during orientation. Curricula on how to use interpreting services have been developed for medical students and could be adapted to the ICU.

While many of these recommendations have been constructed based on existing evidence outside of the ICU setting, adaptation to the ICU is feasible. At present, there is a lack of data for language interventions in the ICU. This gap in knowledge highlights the requirement for a needs analysis and educational/process intervention studies in the critical care setting. There are few examples in the ICU literature of interventions that improve the quality of care, especially for patients with LEP. To combat this, quantitative investigation must be paired with qualitative work to help tailor efforts and provide insight into the needs of patients with LEP in the ICU. Although healthcare staff must constantly balance limited resources in the ICU, care teams must prioritize optimal communication with patients experiencing LEP and their families to ensure equitable care for all.
